# Shared genetic underpinnings of childhood obesity and adult cardiometabolic diseases

**DOI:** 10.1186/s40246-019-0202-x

**Published:** 2019-04-04

**Authors:** Fasil Tekola-Ayele, Anthony Lee, Tsegaselassie Workalemahu, Katy Sánchez-Pozos

**Affiliations:** 1 Epidemiology Branch, Division of Intramural Population Health Research, Eunice Kennedy Shriver National Institute of Child Health and Human Development, National Institutes of Health, 6710B Rockledge Drive, Room 3204, Bethesda, MD 20892-7004 USA; 2 Laboratorio de Endocrinologia Molecular, Hospital Juárez de México, Mexico City, Mexico

**Keywords:** Genetic pleiotropy, Functional loci, Childhood obesity, Cardiometabolic diseases, Developmental Origins of Health and Disease (DoHAD)

## Abstract

**Background:**

Obesity during childhood can lead to increased risk of adverse cardiometabolic diseases such as type 2 diabetes and coronary artery disease during adult life. Evidence for strong genetic correlations between child and adult body mass index (BMI) suggest the possibility of shared genetic effects. We performed a test for pleiotropy (shared genetics) and functional enrichment of single nucleotide polymorphisms (SNPs) associated with childhood BMI and 15 adult cardiometabolic traits using a unified statistical approach that integrates pleiotropy and functional annotation data.

**Results:**

Pleiotropic genetic effects were significantly abundant in 13 out of 15 childhood BMI-adult cardiometabolic trait tests (*P* < 3.3 × 10^−3^). SNPs associated with both childhood BMI and adult traits were more likely to be functionally deleterious than SNPs associated with neither trait. Genetic variants associated with increased childhood obesity tend to increase risk of cardiometabolic diseases in adulthood. We replicated 39 genetic loci that are known to be associated with childhood BMI and adult traits (coronary artery disease, HDL cholesterol, myocardial infarction, triglycerides, total cholesterol, type 2 diabetes, BMI, waist circumference, and waist-to-hip ratio) in previous genome-wide association studies. We also found a novel association of rs12446632 near *GPRC5B*, which is highly expressed in adipose tissue and the central nervous system, with adult HDL cholesterol.

**Conclusions:**

This study found significant pleiotropic genetic effects and enrichment of functional annotations in genetic variants that were jointly associated with childhood obesity and adult cardiometabolic diseases. The findings provide new avenues to disentangle the genetic basis of life course associations between childhood obesity and adult cardiometabolic diseases.

**Electronic supplementary material:**

The online version of this article (10.1186/s40246-019-0202-x) contains supplementary material, which is available to authorized users.

## Background

Obesity in childhood is increasingly becoming a significant global public health burden [1]. Several studies have documented that higher childhood body mass index (BMI), an established measure of obesity, is associated with increased risk of adverse cardiometabolic outcomes in adulthood such as type 2 diabetes, hypertension, dyslipidemia, coronary artery disease, and markers of cardiovascular diseases [2–13]. Identifying common biological pathways underlying childhood adiposity and adult diseases will help unravel mechanisms linking childhood BMI and adult cardiometabolic diseases. It will also provide insights that will help distinguish adiposity-related biological processes that operate in childhood from those that operate in adulthood and to formulate possible causal relationships.

Recent evidence for strong genetic correlations between childhood BMI and a few adulthood cardiometabolic traits such as BMI [14, 15] hint the possible role of genetic pleiotropy, a phenomenon in which a genetic variant(s) influence two or more traits [16–18]. Moreover, single nucleotide polymorphisms (SNPs) associated with adulthood BMI exert their influence on adiposity during childhood [15, 19–21]. Twelve out of 15 SNPs associated with childhood BMI at a genome-wide level of significance are also associated with BMI in adults [15]. Further interrogation of the National Human Genome Research Institute-EBI (NHGRI-EBI) genome-wide association study (GWAS) catalog [22] reveals that some childhood BMI loci are associated with other measures of adulthood adiposity such as hip circumference, waist circumference, body fat distribution, and fat body mass, energy intake, and type 2 diabetes (*ADCY3*, *ZNF646P1*, *MC4R*, *GPR61*, *BRINP2*, *FTO*) [23–27]. These observations highlight the roles of shared genetic effects; however, to date, the extent of genetic pleiotropy between childhood BMI and a wide range of adult cardiometabolic diseases has not been investigated.

In the present study, we performed a comprehensive analysis of genome-wide summary statistics data for childhood BMI and 15 adult cardiometabolic disease traits (hereafter referred to as adult traits) with the following aims: (1) to test for genetic pleiotropy and enrichment of functional loci in childhood BMI and adult trait pairs, (2) to identify genetic variants associated with childhood BMI and an adult trait, and (3) to investigate the regulatory functions of the identified loci and gain additional insights into the underlying common mechanisms and molecular pathways linking childhood BMI and adult traits.

## Results

### Effect of genetic pleiotropy in childhood BMI and adult traits

Our analyses involved childhood BMI and 15 adult traits (BMI, waist-to-hip ratio, waist circumference, type 2 diabetes, fasting plasma glucose, fasting plasma insulin, glycated hemoglobin, insulin secretion, insulin sensitivity, coronary artery disease, myocardial infarction, low-density lipoprotein (LDL) cholesterol, high-density lipoprotein (HDL) cholesterol, total cholesterol, and triglycerides) (Additional file 1: File S1). We observed evidence for genetic pleiotropy between childhood BMI and adult traits for 13 out of 15 adult traits (except fasting plasma glucose and insulin secretion) (empirical *P* < 3.33 × 10^−3^; binomial test *P =* 0.004) (Table 1, Additional file 1: File S2). Variants associated with higher childhood BMI were associated with increased adult BMI, waist circumference, waist-to-hip ratio, triglycerides, type 2 diabetes risk, myocardial infarction risk, and lower HDL.

**Table 1:** Genetic pleiotropy and enrichment of functional deleteriousness among genetic loci associated with childhood BMI and adult cardiometabolic traits

Adult cardiometabolic trait	Genetic pleiotropy			Functional annotation enrichment		
	*π*_11_ (s.e.)	Pleiotropy test statistics	*P* value	*π*_11_ (s.e.)	Annotation test statistics	*P* value
Body mass index	0.055 (0.001)	23,690.45	< 10^− 300^	1.30 (0.04)	173.01	2.85 × 10^−37^
Coronary artery disease	0.01 (0)	1215.77	2.29 × 10^−266^	1.60 (0.13)	87.27	8.47 × 10^−19^
Fasting glucose	0 (0.001)	1.21	0.27	0.04 (2.74)	6.44	0.09
Fasting insulin	0.047 (0.003)	509.74	7.24 × 10^−113^	1.004 (0.22)	41.46	5.23 × 10^−09^
Hemoglobin A1c	0.006 (0)	87.12	1.02 × 10^−20^	2.52 (0.22)	48.73	1.49 × 10^−10^
HDL cholesterol	0.005 (0)	2151.79	< 10^−300^	1.54 (0.12)	88.39	4.86 × 10^−19^
Insulin secretion	0 (0)	0.89	0.35	7.31 (1.79)	38.69	2.01 × 10^−08^
Insulin sensitivity	0.013 (0.001)	393.57	1.38 × 10^−87^	1.54 (0.21)	45.63	6.81 × 10^−10^
LDL cholesterol	0.003 (0)	739.01	9.85 × 10^−163^	1.29 (0.20)	98.53	3.22 × 10^−21^
Myocardial infarction	0.01 (0)	887.48	5.16 × 10^−195^	1.68 (0.14)	58.51	1.22 × 10^−12^
Type 2 diabetes	0.007 (0)	855.26	5.23 × 10^−188^	1.37 (0.19)	65.95	3.14 × 10^−14^
Total cholesterol	0.005 (0)	1186.54	5.14 × 10^−260^	1.45 (0.15)	124.99	6.47 × 10^−27^
Triglycerides	0.003 (0)	1005.09	1.40 × 10^−220^	1.44 (0.17)	74.82	3.97 × 10^−16^
Waist circumference	0.026 (0)	14,809.27	< 10^−300^	1.43 (0.06)	112.69	2.89 × 10^−24^
Waist-to-hip ratio	0.019 (0)	5271.53	< 10^− 300^	1.60 (0.08)	113.88	1.61 × 10^−24^

*π*_11_ is the probability of association of SNPs with both tested traits. *q*_11_/*q*_00_ is the ratio of the probability of jointly associated SNPs being functionally annotated to the probability of a null SNP (associated with neither trait) being functionally annotated

### Enrichment of functional annotations

**Fig. 1:**
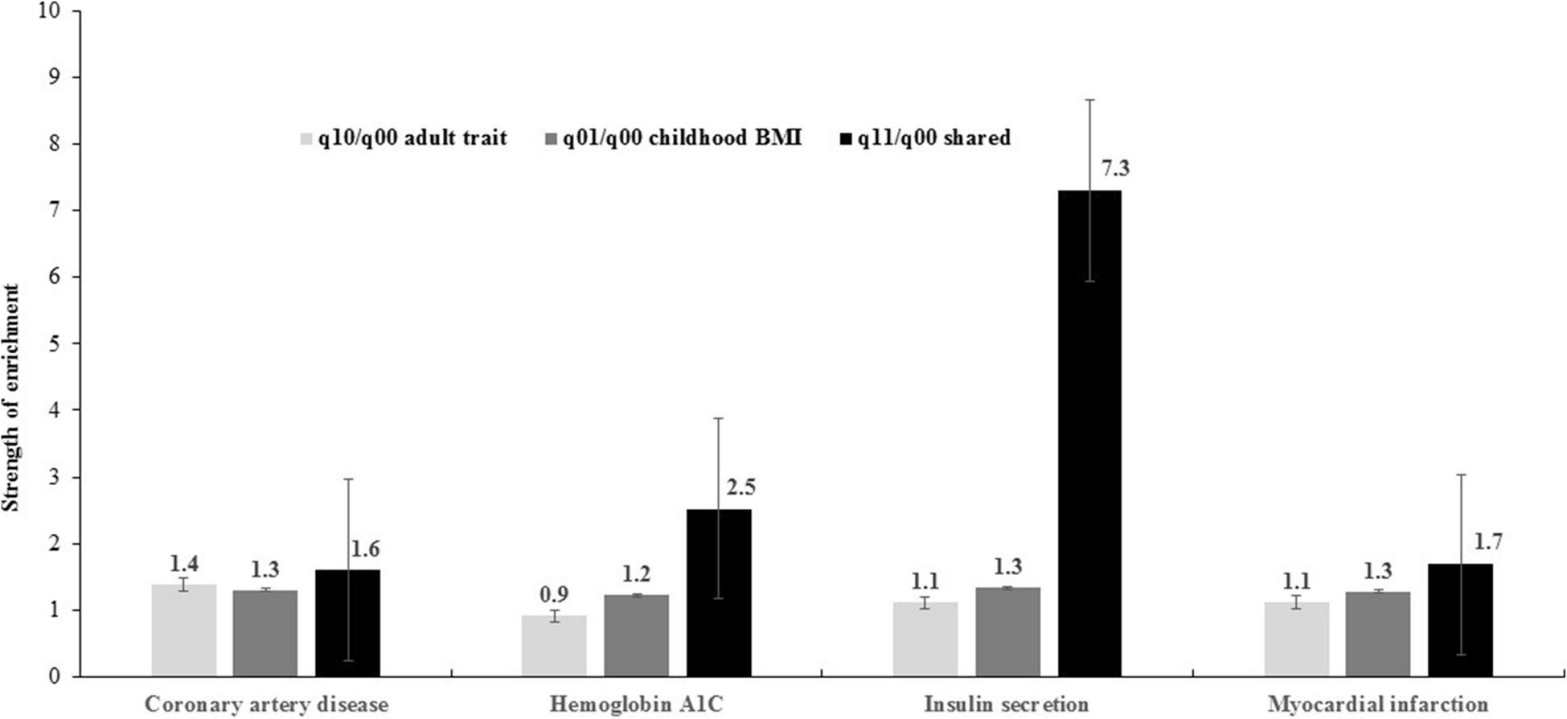
Enrichment of functional annotations for variants associated with childhood BMI and adult cardiometabolic traits. Vertical lines crossing the bars represent standard error. *q*_01_/*q*_00,_ *q*_10_/*q*_00, and_ *q*_11_/*q*_00_ represent the ratio of the probability of SNPs associated with adult traits, child traits, and both traits, respectively, being functionally annotated to the probability of a null SNP being functionally annotated

In 14 out of 15 childhood BMI-adult trait tests, SNPs associated with both childhood BMI and adult traits were more likely to be functionally deleterious than SNPs associated with neither traits (*q*_11_/*q*_00_ ranging from 1.004 to 7.31; *P* < 3.33 × 10^−3^) (Table 1, Additional file 1: File S2). Notably, enrichment of functional deleteriousness was stronger for SNPs associated with both childhood BMI and adult traits than SNPs associated only with childhood BMI or only with adult traits in four childhood BMI-adult trait pairs (coronary artery disease, hemoglobin A1C, insulin secretion, and myocardial infarction). The enrichment folds (*s.e.*) for SNPs associated with childhood BMI-adult trait vs. SNPs associated with adult trait only vs. SNPs associated with childhood BMI only were as follows: 1.60 (0.13) vs. 1.38 (0.12) vs. 1.29 (0.06) for coronary artery disease; 2.52 (0.22) vs. 0.91 (0.23) vs. 1.22 (0.07) for hemoglobin A1C; 7.31 (1.79) vs. 1.11 (0.39) vs. 1.33 (0.05) for insulin secretion; and 1.68 (0.14) vs. 1.11 (0.19) vs. 1.27 (0.06) for myocardial infarction (Fig. 1, Additional file 1: File S2).

### Genetic loci with pleiotropic effects

**Fig. 2:**
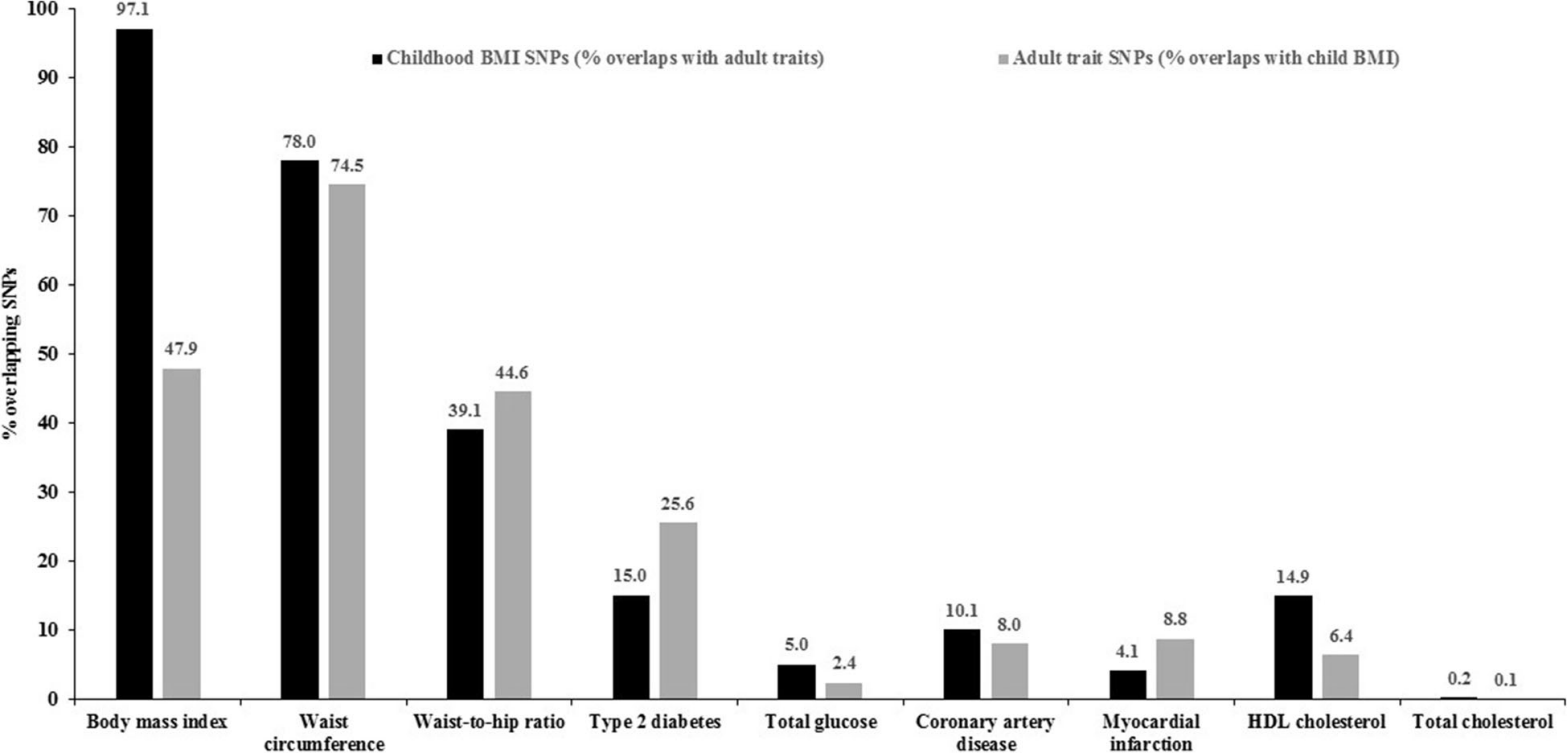
Percentage of SNPs associated with both childhood BMI and adult cardiometabolic traits out of all SNPs associated with both traits

The frequency distribution of SNPs associated with childhood BMI only, with adult traits only, and with both childhood BMI and adult traits is shown in Fig. 2 and Additional file 1: File S3. Out of all SNPs that were significantly associated with either or both childhood BMI and an adult trait, the proportions of SNPs commonly associated with both traits were 61.54% for waist circumference, 47.25% for BMI, 26.32% for waist-to-hip ratio, 10.47% for type 2 diabetes, 4.68% for coronary artery disease, 4.67% for HDL cholesterol, 2.87% for myocardial infarction, 1.68% for triglycerides, and 0.07% for total cholesterol. Of the total number of SNPs associated with childhood BMI, 97.07% were shared with adult BMI. Of the total number of SNPs associated with adult BMI, 47.93% overlapped with childhood BMI (Fig. 2, Additional file 1: File S3).

**Fig. 3:**
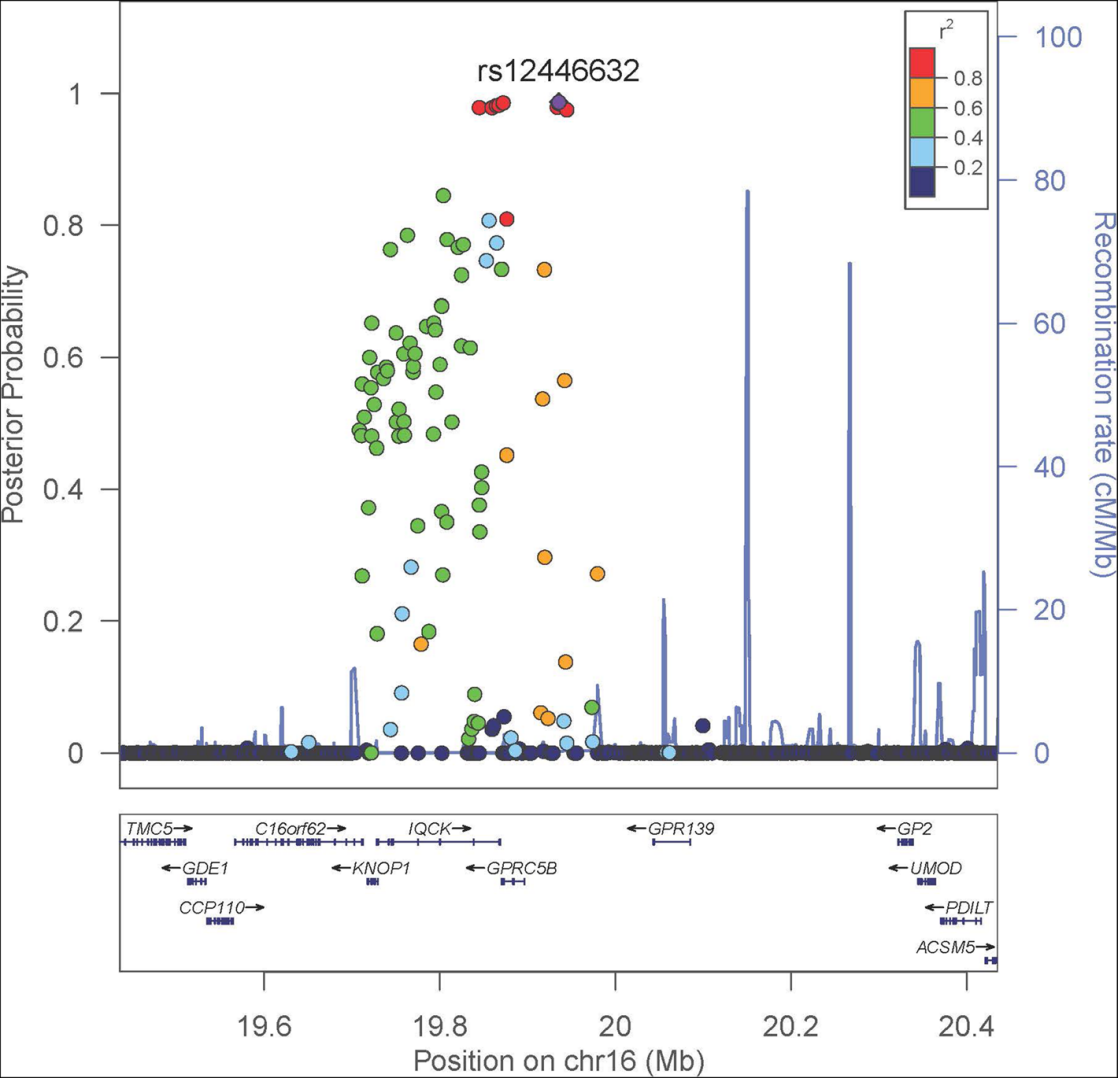
Regional association plot of the *GPRC5B*-*GPR139* locus significantly associated with childhood BMI and adult HDL cholesterol. Data span 500 kb centered at the index SNP rs12446632. The *x*-axis denotes genomic position and the *y*-axis denotes the posterior probability of association and recombination rate (cM/Mb). The purple circle point represents the index SNP. The color of each data point indicates its linkage disequilibrium value (*r*^2^) with the index SNP based on HapMap2

A total of 40 loci were associated with childhood BMI and at least one of the following 9 adult traits: BMI, coronary artery disease, HDL cholesterol, myocardial infarction, type 2 diabetes, total cholesterol, triglycerides, waist circumference, and waist-to-hip ratio (Additional file 1: File S4). Of the 40 loci, 39 loci map to previously known GWAS signals associated with childhood BMI and the adult traits tested (*P* < 5 × 10^−8^ in the NHGRI-EBI GWAS catalog: www.ebi.ac.uk/gwas/). One locus (rs12446632 G, near *GPRC5B*-*GPR139*) significantly associated with higher childhood BMI and lower adult HDL cholesterol in our study (Fig. 3) is a known GWAS locus for childhood BMI [15] but has been only suggestively associated with HDL cholesterol in previous GWAS [28]. In further functional follow-up analysis, we observed that rs12446632 was *cis*-eQTL with expression of the *KNOP1*, *GPRC5B*, and *IQCK* genes in a wide range of tissues (Additional file 1: File S5). The SNP has relatively high functional deleteriousness (CADD = 10.96), and it is within promotor histone marks in gastrointestinal tract mucosa and HepG2 hepatocellular carcinoma cell lines (Haploreg).

### Enrichment for biological pathways and drug ontologies

The set of genes associated with childhood BMI and adult traits were significantly enriched for several biological pathways. The top five most significantly enriched canonical pathways included IL-1 signaling (ratio = 3.26%, *P* = 1.47 × 10^−6^), androgen signaling (ratio = 2.7%, *P* = 2.81 × 10^−6^), corticotropin-releasing hormone signaling (ratio = 2.65%, *P* = 2.97 × 10^−6^), thrombin signaling (ratio = 1.96%, *P* = 6.93 × 10^−7^), and molecular mechanisms of cancer (ratio = 1.27%, *P* = 6.14 × 10^−7^) (Additional file 1: File S6). Ontological analysis found enrichment for disease annotations related to body weight (*P* = 10^−11^; FDR = 1.5 × 10^−8^), obesity (*P* = 1.61 × 10^−7^; FDR = 1.21 × 10^−4^), and schizophrenia (*P* = 5.45 × 10^−5^; FDR = 2.73 × 10^−2^), as well as enrichment for annotations of the drug ontologies related to low energy diets (*P* = 2.26 × 10^−5^; FDR = 2.93 × 10^−2^) (Additional file 1: File S7).

## Discussion

The present study comprehensively assessed genetic pleiotropic effects in childhood BMI and adult cardiometabolic diseases and showed evidence for shared genetic influence in childhood adiposity and adult chronic diseases. The study also found that SNPs with known biological functions are more likely to be associated with both childhood BMI and adult traits compared to SNPs that are not functional. We replicated 39 genetic loci that are known to be associated with childhood BMI and adult traits in previous GWAS. Additionally, we found novel association of rs12446632 in the *GPRC5B*-*GPR139* locus with adult HDL cholesterol. In all, the findings of the study provide evidence for common genetic mechanisms underlying childhood adiposity and development of cardiometabolic outcomes, thereby facilitating discovery of therapeutic and preventive targets to improve cardiometabolic health across the life span.

Although no genome-wide evaluation of genetic pleiotropy in childhood BMI and a range of adult traits has been done, cross-trait evaluation of individual GWAS loci and genetic risk scores derived from the loci presented in this study have been evaluated in relation to childhood and adulthood BMI. These studies have found that adult BMI loci also operate during childhood [20, 29–33]. Out of the 97 known adult BMI SNPs discovered in a more recent large-scale GWAS, 86 SNPs had directionally similar effect on childhood BMI and 50 were nominally associated with childhood BMI [15]. The two most recent childhood BMI GWAS studies have reported that seven out of eight loci [34], and 12 out of 15 loci associated with childhood BMI [15], are also associated with BMI in adults. Meanwhile, a strong genetic correlation between childhood BMI and adult BMI has been observed (*ρ* = 0.73) [15]. A few other studies have also reported genetic loci that overlap in their associations with childhood BMI or obesity, adult type 2 diabetes (*FTO*, *ID*-*HHEX*) [26, 35], bone mineral density (*SP7*) [36], waist circumference (*TNKS*-*MSRA*) [37], and triglyceride levels (*TNKS*-*MSRA*) [38].

Our study found several SNPs that overlapped in their associations with childhood BMI and adult adiposity traits, type 2 diabetes, coronary artery disease, and HDL cholesterol. Notably, the overwhelming majority of childhood BMI genetic loci continue to exert influence on adult BMI. Therefore, genetic factors may partly explain the widely known observation that childhood BMI tracks through adulthood [39]. Our finding also strengthens previous observations of substantial overlaps in the genetic architecture of childhood and adulthood obesity [20, 29–33]. Furthermore, we found that the genetic architecture of childhood BMI is mirrored to a large extent by adult waist circumference (61.54% overlapping SNPs associated with childhood BMI) and BMI (47.25% overlapping SNPs) but to a lesser extent by waist-to-hip ratio (26.32% overlapping SNPs). Similarly, previous studies have shown that a higher adult BMI genetic risk score [19, 20], but not a higher waist-to-hip ratio genetic risks score [20], is associated with higher childhood BMI. Future studies interrogating the influence of adult waist circumference genetic loci on childhood adiposity will have the potential to provide new insights into mechanistic underpinnings of the early origins of total body and visceral adiposity.

The association of rs12446632 (near *GPRC5B*) with childhood BMI-adult HDL cholesterol in our study is noteworthy. A previous GWAS has already reported its association with childhood BMI [15]. The novelty of our finding is the association of rs12446632 with adult HDL cholesterol; the SNP or its proxies (in  strong linkage-disequilibrium) fell short of genome-wide significance in previous GWAS of HDL cholesterol [28]. The allele associated with increased childhood BMI was also associated with lower HDL cholesterol, consistent with observational studies that found inverse correlations between childhood BMI and adult HDL cholesterol levels [4, 40]. SNP rs12446632 may have important functional roles given its proximity (39 kbp) to the 5′ of *GPRC5B*, its relatively high CADD deleteriousness score, and evidence of roles in regulating expression of *GPRC5B*, and overlapping histone marks. GPRC5B is highly expressed in adipose tissue and the central nervous system [41]. The encoded protein is a lipid raft-associated transmembrane protein that may be critical for inflammatory signaling in adipose tissue [41, 42] and modulates insulin secretion [43]. We also observed that rs12446632 was associated with adulthood adiposity measures consistent with previous GWAS that reported significant associations of the SNP with adulthood BMI [21, 44, 45], obesity [46], waist circumference [23], and hip circumference [23]. Given the known association of adulthood BMI with dyslipidemia [47] and this widely replicated association of the SNP with obesity [21, 23, 44–46], it will be noteworthy to investigate whether the rs12446632-adult HDL cholesterol association found in our study is mediated through BMI during adulthood.

Our findings demonstrating significant enrichment of pathways such as IL-1 signaling, androgen signaling, corticotropin-releasing hormone signaling, and thrombin signaling highlight the possibility of the relationships between childhood adiposity and adulthood cardiometabolic diseases. These relationships may involve mechanisms broader than endothelial dysfunction, insulin resistance, inflammation, and adipocytokines [48, 49]. More detailed understanding of the pathways in which childhood adiposity and adult cardiometabolic disease traits overlap could provide new avenues for therapeutic targeting. This seems promising given our observation of enrichment for metabolic disease-related ontologies and potential drug targets among the set of genes jointly associated with childhood BMI and adult traits. Furthermore, our study showed that genetic variants associated with increased childhood adiposity tend to increase the risk of obesity, cardiovascular diseases, type 2 diabetes, and dyslipidemia in adulthood. This finding suggests that prevention of childhood obesity informed by genetic evidence will be beneficial to lower cardiometabolic disease risk in later life.

We acknowledge that there are limitations to our study. Despite the large sample sizes of the consortia-based meta-analysis studies, there were differences in sample size and number of SNPs among the different studies. As a result, the traits for which the source studies had relatively fewer loci and samples were likely to be less enriched for SNPs with potential shared influence (e.g., fasting glucose). In addition, some of the observed associations may not be due to independent effects of the same locus on childhood BMI and an adult trait, but due to the correlation of traits that are in the causal pathway or through other unmeasured traits. An important strength of our study is the integrated modeling of functional annotation and GWAS summary statistics data from pairs of traits. This multi-trait approach has been instrumental in testing for functional enrichment and detection of novel loci with multi-trait effects [50]. Implementation of this approach considerably expanded our understanding of the genetic links between childhood BMI and adult traits.

## Conclusions

The present study found pleiotropic genetic effects on childhood obesity and adult cardiometabolic diseases. The previously identified genetic loci, including our novel loci with pleiotropic effects, were functionally enriched for biological pathways related to adiposity and cardiovascular diseases. These biological pathways through which the genes operate provide the potential to disentangle the genetic basis of life course associations between childhood obesity and adult cardiometabolic diseases.

## Methods

### Data sets

We assembled GWAS summary statistics data including *P* values and effect directions of genome-wide SNPs reported by six consortia [21, 23, 28, 51–57], involving childhood BMI and 15 adult traits (BMI, waist-to-hip ratio, waist circumference, type 2 diabetes, fasting plasma glucose, fasting plasma insulin, glycated hemoglobin, insulin secretion, insulin sensitivity, coronary artery disease, myocardial infarction, LDL cholesterol, HDL cholesterol, total cholesterol, and triglycerides). The majority of the study participants had European ancestry, with some studies additionally involving individuals of East Asian, South Asian, and Hispanic- and African-Americans (Additional file 1: File S1).

### Functional annotation of SNPs

Functional annotation of SNPs was carried out using the Combined Annotation Dependent Depletion (CADD) framework as implemented in CADD v1.2 (http://cadd.gs.washington.edu) [58]. CADD integrates functional and evolutionary importance from multiple annotation sources into one metric by contrasting variants that survived natural selection with simulated mutations to generate a deleteriousness score for each genetic variant. Variants with Phred-like CADD score (− 10*log10 [rank/total]) values ≥ 15 were considered deleterious [58] and were assigned annotation of 1, and those with CADD score values < 15 were assigned annotation of 0. The assigned annotation values were used as inputs in annotation tests. Subsequent annotation tests assessed functional enrichment among (1) SNPs associated only with childhood BMI compared to SNPs associated with neither trait (estimated by *q*_10_/*q*_00_), (2) SNPs associated only with an adult trait compared to SNPs associated with neither trait (*q*_01_/*q*_00_), and (3) SNPs associated with both childhood BMI and an adult trait compared to SNPs associated with neither trait (*q*_11_/*q*_00_). *q*_11_/*q*_00_ is the ratio of the probability of jointly associated SNPs being functionally annotated to the probability of a null SNP (associated with neither trait) being functionally annotated [50].

### Tests for genetic pleiotropy

We tested for evidence of pleiotropy, enrichment of functional annotation, and association of SNPs with both childhood BMI and an adult trait using the GPA v1.1-0 R package [50]. GPA (Genetic analysis incorporating Pleiotropy and Annotation) implements a unified statistical approach that integrates pleiotropy and functional annotation data and tests for enrichment of annotations from functional datasets in variants associated with pairs of traits. A total of 15 childhood BMI-adult trait pair tests were performed.

All tests were conducted under the false discovery rate control (FDR) at the 0.05 level using 10,000 expectation maximization iterations. Evidence for enrichment of pleiotropy and functional annotation were considered significant at the Bonferroni-corrected level *P* value = 3.33 × 10^−3^ (0.05/15 tests). SNPs were considered to be significantly associated with both traits in a childhood BMI-adult trait pair if posterior probability of association was > 0.95 with FDR of 0.05 as implemented in GPA [50]. When two or more SNPs within a 1 Mb region were associated with a trait pair, the index SNP with the highest posterior probability of association and other SNPs not in LD with the index SNP (*r*^2^ < 0.06 in the 1000 Genomes Phase 3 CEU population sample) were considered to be independent associations. SNPs that were newly identified to be associated with childhood BMI and/or adult traits were examined for potential regulatory effect on gene expression level in different tissues using the Genotype-Tissue Expression (GTEx v. 6) [59] database. Possible regulatory effects of the lead SNPs were assessed by examining if the SNPs are within promoters, enhancers, DNAse, and transcription factor binding using the Haploreg tool (version 4.1) [60].

### Ontology analysis and drug target annotations

We performed ontology analysis on the list of genes mapping to loci associated with childhood BMI-adult trait for connectivity using the online tool WEB-based GEne SeT AnaLysis Toolkit (WebGestalt) [61]. The hypergeometric distribution was used to test for statistical significance. Adjustment for multiple testing was controlled using the Benjamini-Hochberg procedure.

### Pathway analysis

To determine whether the list of genes showing significant association with childhood BMI-adult traits was enriched in biological functions or processes relevant to those traits, we looked for enrichment of biological pathways using the Ingenuity Pathway Analysis (IPA) bioinformatics resource (IPA, Qiagen, Redwood City, CA, USA). We examined the overlap of the pleiotropic gene list with gene sets representing canonical pathways in IPA for the genes associated with childhood BMI and adult traits. Fisher’s exact test was used to assess the statistical significance of the overlap between our list of pleiotropic genes submitted to IPA and the list of genes in canonical pathways in the databases.

## Additional file

Additional file 1: **File S1.** GWAS summary statistics profile of traits and diseases analyzed in the study. **File S2.** Genetic pleiotropic effects and enrichment of functionally deleterious SNPs associated with childhood BMI-adult cardiometabolic traits. **File S3.** Number of SNPs associated only with childhood BMI, only with an adult cardiometabolic trait, and both childhood BMI and an adult cardiometabolic trait with posterior probability > 0.95. **File S4.** Genetic loci significantly associated with both childhood BMI and adult cardiometabolic traits with posterior probability > 0.95. **File S5.** Genes whose expression levels were significantly associated with SNP rs12446632. **File S6.** Canonical pathways significantly enriched in genes associated with childhood BMI-adult cardiometabolic traits. **File S7.** Significantly over-represented disease and drug ontologies in the set of genes associated with childhood BMI-adult cardiometabolic traits. (DOCX 282 kb)

## Abbreviations

BMI: Body mass index
CADD: Combined Annotation Dependent Depletion
eQTL: Expression quantitative trait locus
GPA: Genetic analysis incorporating Pleiotropy and Annotation
GTEx: Genotype-Tissue Expression
GWAS: Genome-wide association study
HDL: High-density lipoprotein
IPA: Ingenuity Pathway Analysis
LD: Linkage disequilibrium
LDL: Low-density lipoprotein
SNP: Single nucleotide polymorphism
WebGestalt: WEB-based GEne SeT AnaLysis Toolkit
