# Supplementary material for: Shared genetic underpinnings of childhood obesity and adult cardiometabolic diseases
Source: Hum Genomics. 2019 Apr 4;13:17. doi: 10.1186/s40246-019-0202-x (PMC6449964; doi:10.1186/s40246-019-0202-x)
Supplement: Supplementary file 1 — File S1. GWAS summary statistics profile of traits and diseases analyzed in the study. File S2. Genetic pleiotropic effects and enrichment of functionally deleterious SNPs associated with childhood BMI-adult cardiometabolic traits. File S3. Number of SNPs associated only with childhood BMI, only with an adult cardiometabolic trait, and both childhood BMI and an adult cardiometabolic trait with posterior probability > 0.95. File S4. Genetic loci significantly associated with both childhood BMI and adult cardiometabolic traits with posterior probability > 0.95. File S5. Genes whose expression levels were significantly associated with SNP rs12446632. File S6. Canonical pathways significantly enriched in genes associated with childhood BMI-adult cardiometabolic traits. File S7. Significantly over-represented disease and drug ontologies in the set of genes associated with childhood BMI-adult cardiometabolic traits. (DOCX 282 kb) [file 40246_2019_202_MOESM1_ESM.docx]

**Additional File**

**Shared genetic underpinnings of childhood obesity and adult cardiometabolic diseases**

Fasil Tekola-Ayele^1*^, Anthony Lee^1^, Tsegaselassie Workalemahu^1^, Katy Sánchez-Pozos^2^

^1^ Epidemiology Branch, Division of Intramural Population Health Research, *Eunice Kennedy Shriver* National Institute of Child Health and Human Development, National Institutes of Health, Bethesda, MD, USA

^2^ Laboratorio de Endocrinologia Molecular, Hospital Juárez de México, Ciudad de México, México

*Correspondence:*

*Fasil Tekola-Ayele, PhD

Epidemiology Branch, Division of Intramural Population Health Research,

*Eunice Kennedy Shriver* National Institute of Child Health and Human Development

National Institutes of Health. 6710B Rockledge Drive, Room 3204, Bethesda, MD 20892-7004

E-mail: ayeleft@mail.nih.gov Tel: 301-827-6518

**Additional File 1.** GWAS summary statistics profile of traits and diseases analyzed in the study.

This Table summarizes the sources of the GWAS summary statistics data used in our paper, the overall sample size, number of SNPs for which summary statistics are available, and publications that describe the studies.

| **Trait/Disease** | **Source/Consortium** | **Sample size** | **Ancestries of GWAS study populations** | **#SNPs** | **PubMed ID (Reference)** |
| --- | --- | --- | --- | --- | --- |
| Childhood BMI | Early Growth Genetics Consortium (EGG);  http://egg-consortium.org | 35,668 | European | 2499453 | 26604143 ((1)) |
| Adult body mass index | Genetic Investigation of Anthropometric Traits (GIANT); <http://portals.broadinstitute.org/collaboration/giant/index.php/GIANT_consortium> | 339,224 | European | 2555510 | 25673413 ((2)) |
| Waist-to-hip ratio |  | 224,459 | European, east Asian, south Asian, African-American | 2598692 | 25673412 ((3)) |
| Waist circumference |  | 224,459 | European, east Asian, south Asian, African-American | 2601994 | 25673412 ((3)) |
| Type 2 diabetes | DIAbetes Genetics Replication And Meta-analysis (DIAGRAM);  <http://www.diagram-consortium.org> | 110,452 | European, east Asian, south Asian, Mexican, African-American | 2914607 | 24509480 ((4)) |
| Fasting glucose | Meta-Analysis of Glucose and Insulin-related traits Consortium (MAGIC);  <https://www.magicinvestigators.org/> | 46,186 | European | 79157 | 20081858 ((5)) |
| Fasting insulin |  | 46,186 | European | 2523878 | 20081858 ((5)) |
| Hemoglobin A1C |  | 46,368 | European | 2598230 | 20858683 ((6)) |
| Insulin secretion – CIR |  | 5,318 | European | 2488776 | 24699409 ((7)) |
| Insulin sensitivity – ISI |  | 16,753 | European | 2485212 | 27416945 ((8)) |
| Coronary artery disease | Coronary Artery Disease Genomewide Replication and Meta-analysis (CARDIoGRAM) plus The Coronary Artery Disease (C4D) Genetics Consortium;  <http://www.cardiogramplusc4d.org/> | 184,305 | European, south Asian, east Asian; smaller samples of Hispanic and African Americans | 9455778 | 26343387 ((9)) |
| Myocardial infarction |  | ~129,000 | European, south Asian, east Asian; smaller samples of Hispanic and African Americans | 11169182 | 26343387 ((9)) |
| LDL cholesterol | Global Lipids Genetics Consortium;  <http://lipidgenetics.org> | 188,577 | European, east Asian, south Asian, African | 2437751 | 24097068 ((10)) |
| HDL cholesterol |  | 188,577 | European, east Asian, south Asian, African | 2447441 | 24097068 ((10)) |
| Total cholesterol |  | 188,577 | European, east Asian, south Asian, African | 2446981 | 24097068 ((10)) |
| Triglycerides |  | 188,577 | European, east Asian, south Asian, African | 2439432 | 24097068 ((10)) |

**Additional File 2.** Genetic pleiotropic effects and enrichment of functionally deleterious SNPs associated with childhood BMI-adult cardiometabolic traits.

| **Adult trait** | **# SNPs** | **pi00 (se)** | **pi10 (se)** | **pi01 (se)** | **pi11 (se)** | **Pleiotropy**  **test statistics** | **P-value** | **q10/q00 (se)** | **q01/q00 (se)** | **q11/q00 (se)** | **Annotation**  **test statistics** | **P-value** |
| --- | --- | --- | --- | --- | --- | --- | --- | --- | --- | --- | --- | --- |
| BMI | 2529650 | 0.936 (0.001) | 0.007(0.001) | 0.001(0.001) | 0.055(0.001) | 23690.447 | < 10^-300^ | 2.351(0.196) | 4.11(0.901) | 1.297(0.041) | 173.008 | 2.85E-37 |
| Coronary artery disease | 2491268 | 0.923 (0.001) | 0.014(0) | 0.053(0.001) | 0.01(0) | 1215.765 | 2.29E-266 | 1.384(0.121) | 1.295(0.062) | 1.599(0.126) | 87.266 | 8.47E-19 |
| Fasting glucose | 78084 | 0.915(0.005) | 0.023(0.003) | 0.062(0.005) | 0(0.001) | 1.207 | 0.2718327 | 0.037(36.904) | 1.685(0.4) | 0.037(2740.429) | 6.435 | 0.0922562 |
| Fasting insulin | 2497167 | 0.885(0.004) | 0.047(0.004) | 0.021(0.003) | 0.047(0.003) | 509.735 | 7.24E-113 | 1.475(0.224) | 2.18(0.268) | 1.004(0.218) | 41.457 | 5.23E-09 |
| Hemoglobin A1C | 2508081 | 0.913(0.001) | 0.018(0.001) | 0.063(0.001) | 0.006(0) | 87.118 | 1.02E-20 | 0.906(0.225) | 1.221(0.067) | 2.52(0.222) | 48.729 | 1.49E-10 |
| HDL cholesterol | 2446597 | 0.931(0.001) | 0.002(0) | 0.062(0.001) | 0.005(0) | 2151.794 | < 10^-300^ | 1.599(0.23) | 1.302(0.052) | 1.536(0.121) | 88.389 | 4.86E-19 |
| Insulin secretion | 2459537 | 0.924(0.001) | 0.005(0) | 0.071(0.001) | 0(0) | 0.887 | 0.3463089 | 1.106(0.389) | 1.329(0.049) | 7.307(1.789) | 38.697 | 2.01E-08 |
| Insulin sensitivity | 2448641 | 0.926(0.001) | 0.004(0.001) | 0.057(0.001) | 0.013(0.001) | 393.572 | 1.38E-87 | 1.77(0.623) | 1.302(0.08) | 1.541(0.205) | 45.626 | 6.81E-10 |
| LDL cholesterol | 2437897 | 0.925(0.001) | 0.003(0) | 0.068(0.001) | 0.003(0) | 739.008 | 9.85E-163 | 1.985(0.131) | 1.312(0.049) | 1.289(0.198) | 98.528 | 3.22E-21 |
| Myocardial infarction | 2504226 | 0.922(0.001) | 0.012(0) | 0.057(0.001) | 0.01(0) | 887.483 | 5.16E-195 | 1.114(0.191) | 1.272(0.062) | 1.684(0.136) | 58.508 | 1.22E-12 |
| Type 2 diabetes | 2507170 | 0.925(0.001) | 0.009(0) | 0.059(0.001) | 0.007(0) | 855.258 | 5.23E-188 | 1.579(0.149) | 1.359(0.055) | 1.366(0.19) | 65.95 | 3.14E-14 |
| Total cholesterol | 2446200 | 0.924(0.001) | 0.005(0) | 0.067(0.001) | 0.005(0) | 1186.54 | 5.14E-260 | 1.788(0.124) | 1.308(0.05) | 1.445(0.15) | 124.997 | 6.47E-27 |
| Triglycerides | 2438985 | 0.93(0.001) | 0.003(0) | 0.065(0.001) | 0.003(0) | 1005.092 | 1.40E-220 | 1.682(0.155) | 1.303(0.051) | 1.439(0.167) | 74.815 | 3.97E-16 |
| Waist circumference | 2522518 | 0.955(0.001) | 0(0) | 0.019(0.001) | 0.026(0) | 14809.27 | < 10^-300^ | 31.721(9.721) | 1.358(0.122) | 1.425(0.056) | 112.69 | 2.89E-24 |
| Waist-to-hip ratio | 2520728 | 0.937(0.001) | 0.001(0) | 0.043(0.001) | 0.019(0) | 5271.527 | < 10^-300^ | 3.089(0.811) | 1.236(0.078) | 1.604(0.078) | 113.875 | 1.61E-24 |

q_01_/q_00 ,_ q_10_/q_00 , and_ q_11_/q_00_ represent the ratio of the probability of SNPs associated with adult traits, child traits, and both traits, respectively, being functionally annotated to the probability of a null SNP being functionally annotated.

**Additional File 3.** Number of SNPs associated only with childhood BMI, only with an adult cardiometabolic trait, and both childhood BMI and an adult cardiometabolic trait with posterior probability >0.95.

| **Adult trait** | **#SNPs**  **Tested** | **10 (associated with an adult trait but not with childhood BMI)** | **01 (associated with childhood BMI but not an adult trait)** | **11 (associated with both childhood BMI and an adult trait)** | **Associated with childhood BMI as % of all SNPs associated with an adult trait (10/[10+11]*100)** | **Associated with an adult trait as % of all SNPs associated with childhood BMI (01/[01+11]*100)** | **Associated with both childhood BMI and an adult trait as % of all SNPs associated with both traits (11/[01+10+11]*100)** |
| --- | --- | --- | --- | --- | --- | --- | --- |
| BMI | 2529650 | 793 | 22 | 730 | 47.93 | 97.07 | 47.25 |
| Coronary artery disease | 2491268 | 640 | 501 | 56 | 8.05 | 10.05 | 4.68 |
| Fasting glucose | 78084 | 9 | 22 | 0 | 0 | 0 | 0 |
| Fasting insulin | 2497167 | 0 | 0 | 0 | NA | NA | NA |
| Hemoglobin A1C | 2508081 | 135 | 659 | 0 | 0 | 0.00 | 0 |
| HDL cholesterol | 2446597 | 1797 | 696 | 122 | 6.36 | 14.91 | 4.67 |
| Insulin secretion | 2459537 | 13 | 891 | 0 | 0 | 0 | 0 |
| Insulin sensitivity | 2448641 | 7 | 771 | 0 | 0 | 0 | 0 |
| LDL cholesterol | 2437897 | 1280 | 918 | 0 | 0 | 0 | 0 |
| Myocardial infarction | 2504226 | 250 | 561 | 24 | 8.76 | 4.10 | 2.87 |
| Type 2 diabetes | 2507170 | 305 | 593 | 105 | 25.61 | 15.04 | 10.47 |
| Total cholesterol | 2446200 | 1930 | 892 | 2 | 0.10 | 0.22 | 0.07 |
| Triglycerides | 2438985 | 1595 | 753 | 40 | 2.45 | 5.04 | 1.68 |
| Waist circumference | 2522518 | 170 | 140 | 496 | 74.47 | 77.99 | 61.54 |
| Waist-to-hip ratio | 2520728 | 292 | 366 | 235 | 44.59 | 39.10 | 26.32 |

**Additional File 4.** Genetic loci significantly associated with both childhood BMI and adult cardiometabolic traits with posterior probability > 0.95.

| **Adult trait** | **SNP** | **Chr** | **pos_37** | **Gene** | **GWAS P-value, adult trait** | **GWAS**  **P-value, childhood BMI** | **PP (00)** | **PP (10) (Adult trait)** | **PP (01) (childhood BMI)** | **PP (11)(childhood BMI-adult trait)** |
| --- | --- | --- | --- | --- | --- | --- | --- | --- | --- | --- |
| BMI | rs2590942 | 1 | 72885281 | *NEGR1* | 8.433e-20 | 3.88E-9 | 8.95877E-16 | 0.001001179 | 8.93926E-13 | 0.999 |
|  | rs12041852 | 1 | 75003500 | *TNNI3K* | 4.398e-15 | 1.771E-10 | 4.38461E-13 | 0.000194844 | 2.24988E-09 | 0.999 |
|  | rs7550711 | 1 | 110082886 | *GNAT2* | 5.059e-14 | 1.504E-8 | 2.68623E-11 | 0.00205211 | 1.30632E-08 | 0.998 |
|  | rs543874 | 1 | 177889480 | *BRINP2, SEC16B* | 2.287e-40 | 2.376E-17 | 5.24421E-28 | 4.40637E-08 | 1.19014E-20 | 0.999 |
|  | rs4854349 | 2 | 647861 | *FAM150B, TMEM18* | 2.336e-51 | 5.996E-21 | 6.47147E-30 | 5.43755E-10 | 1.19014E-20 | 0.999 |
|  | rs17734264 | 2 | 24719710 | *ADCY3* | 4.323e-06 | 3.659E-9 | 6.58693E-06 | 0.000963954 | 0.006780276 | 0.992 |
|  | rs11895026 | 2 | 25036858 | *CENPO* | 2.426e-06 | 4.233E-11 | 4.09083E-07 | 9.07916E-05 | 0.00448512 | 0.995 |
|  | rs13130484 | 4 | 45175691 | *PRDX4P1, PRKRIRP9* | 8.011e-41 | 8.936E-11 | 1.61331E-24 | 0.000135556 | 1.18998E-20 | 0.999 |
|  | rs13107325 | 4 | 103188709 | *SLC39A8, NFKB1* | 1.064e-12 | 1.194E-8 | 2.13639E-10 | 0.001816041 | 1.17426E-07 | 0.998 |
|  | rs4569924 | 5 | 153540025 | *MFAP3, GALNT10* | 2.799e-08 | 4.056E-7 | 2.10754E-06 | 0.011667058 | 0.0001785 | 0.988 |
|  | rs987237 | 6 | 50803050 | *TFAP2B* | 1.074e-30 | 3.809E-13 | 9.3907E-26 | 7.4946E-06 | 1.25299E-20 | 0.999 |
|  | rs3829849 | 9 | 129390800 | *LMX1B* | 4.994e-07 | 1.457E-6 | 3.27393E-05 | 0.022705632 | 0.001407085 | 0.976 |
|  | rs17309930 | 11 | 27748493 | *BDNF-AS* | 1.773e-14 | 2.474E-8 | 1.64169E-11 | 0.002670526 | 6.13101E-09 | 0.997 |
|  | rs7132908 | 12 | 50263148 | *NCKAP5L* | 3.515e-14 | 4.985E-19 | 5.71184E-17 | 5.67323E-09 | 1.00681E-08 | 0.999 |
|  | rs12429545 | 13 | 54102206 | *ZNF646P1, LINC00558* | 3.152e-13 | 3.656E-11 | 4.12975E-12 | 8.43801E-05 | 4.89381E-08 | 0.999 |
|  | rs10151686 | 14 | 30466466 | *PRKD1, RPS6P24* | 1.291e-06 | 1.503E-6 | 6.58858E-05 | 0.023041799 | 0.002785363 | 0.974 |
|  | rs8046312 | 16 | 19979334 | *GPRC5B, GPR139* | 1.351e-09 | 4.062E-10 | 6.1488E-09 | 0.000302609 | 2.03127E-05 | 0.999 |
|  | rs1421085 | 16 | 53800954 | *FTO* | 2.17e-158 | 3.201E-19 | 5.33797E-29 | 4.48514E-09 | 1.19014E-20 | 0.999 |
|  | rs6567160 | 18 | 57829135 | *PMAIP1-MC4R* | 6.684e-59 | 4.062E-12 | 3.1307E-25 | 2.63052E-05 | 1.19011E-20 | 0.999 |
| Coronary artery disease | rs571312 | 18 | 57839769 | *PMAIP1-MC4R* | 3.88e-08 | 7.176E-12 | 2.09111E-08 | 3.20062E-05 | 0.0006529 | 0.999 |
| HDL cholesterol | rs17024393 | 1 | 110154688 | *GNAT2* | 5.084E-6 | 3.076E-8 | 7.06557E-05 | 0.002890282 | 0.023791951 | 0.973 |
|  | rs13135688 | 4 | 103285906 | *SLC39A8, NFKB1* | 8.238E-9 | 5.065E-8 | 2.65584E-07 | 0.003851938 | 6.86779E-05 | 0.996 |
|  | rs12446632 | 16 | 19935389 | *GPRC5B, GPR139* | 2.407E-6 | 1.321E-9 | 6.83715E-06 | 0.000553843 | 0.012187559 | 0.987 |
|  | rs1121980 | 16 | 53809247 | *FTO* | 6.791E-9 | 6.122E-19 | 3.70861E-13 | 6.41712E-09 | 5.77891E-05 | 0.999 |
|  | rs6567160 | 18 | 57829135 | *PMAIP1-MC4R* | 2.918E-9 | 4.062E-12 | 7.00839E-10 | 2.62392E-05 | 2.67082E-05 | 0.999 |
| Myocardial infarction | rs663129 | 18 | 57838401 | *PMAIP1-MC4R* | 1.73E-06 | 9.771E-11 | 2.30611E-06 | 0.000137567 | 0.016484859 | 0.983 |
| Type 2 diabetes | rs10190052 | 2 | 646674 | *FAM150B, TMEM18* | 7.5E-6 | 5.997E-18 | 9.10002E-10 | 2.03669E-08 | 0.042769402 | 0.957 |
|  | rs3751812 | 16 | 53818460 | *FTO* | 6.7E-15 | 2.939E-18 | 1.7586E-16 | 1.45781E-08 | 1.20633E-08 | 0.999 |
|  | rs571312 | 18 | 57839769 | *PMAIP1-MC4R* | 2.1E-8 | 7.176E-12 | 2.22275E-08 | 3.54715E-05 | 0.000626215 | 0.999 |
| Total cholesterol | rs7550711 | 1 | 110082886 | *GNAT2* | 2.58E-7 | 1.504E-8 | 2.41445E-06 | 0.002024953 | 0.001188516 | 0.997 |
| Triglycerides | rs9930333 | 16 | 53799977 | *FTO* | 3.251E-8 | 2E-13 | 1.53458E-09 | 5.32122E-06 | 0.000288305 | 0.999 |
|  | rs2168708 | 18 | 57907311 | *PMAIP1-MC4R* | 6.196E-6 | 6.157E-8 | 0.000149657 | 0.004108705 | 0.034994687 | 0.961 |
| Waist circumference | rs2590942 | 1 | 72885281 | *NEGR1* | 9.1e-11 | 3.88E-9 | 4.1205E-09 | 0.000999829 | 4.11706E-06 | 0.999 |
|  | rs12566985 | 1 | 75002193 | *TNNI3K* | 2e-08 | 2.207E-10 | 5.34551E-08 | 0.000218707 | 0.000244301 | 0.999 |
|  | rs7550711 | 1 | 110082886 | *GNAT2* | 3.1e-09 | 1.504E-8 | 1.22079E-07 | 0.00204885 | 5.94587E-05 | 0.998 |
|  | rs543874 | 1 | 177889480 | *BRINP2,SEC16B* | 2e-26 | 2.376E-17 | 2.54148E-25 | 4.41192E-08 | 5.76048E-18 | 0.999 |
|  | rs13397165 | 2 | 653354 | *FAM150B, TMEM18* | 7.6e-31 | 2.685E-20 | 3.85414E-30 | 1.20711E-09 | 3.19286E-21 | 0.999 |
|  | rs11676272 | 2 | 25141538 | *ADCY3* | 5.2e-09 | 8.548E-23 | 5.04192E-15 | 5.71991E-11 | 8.81391E-05 | 0.999 |
|  | rs13130484 | 4 | 45175691 | *PRDX4P1, PRKRIRP9* | 9.3e-22 | 8.936E-11 | 2.66632E-18 | 0.000135444 | 1.96831E-14 | 0.999 |
|  | rs7715256 | 5 | 153537893 | *MFAP3, GALNT10* | 1e-05 | 4.852E-7 | 0.000336417 | 0.012456095 | 0.025961577 | 0.961 |
|  | rs987237 | 6 | 50803050 | *TFAP2B* | 9.7e-19 | 3.809E-13 | 2.84442E-17 | 7.49404E-06 | 3.79554E-12 | 0.999 |
|  | rs17309930 | 11 | 27748493 | *BDNF-AS* | 5.2e-12 | 2.474E-8 | 1.25844E-09 | 0.002666262 | 4.70727E-07 | 0.997 |
|  | rs7138803 | 12 | 50247468 | *NCKAP5L* | 1.6e-16 | 4.978E-18 | 3.48858E-18 | 1.9259E-08 | 1.8114E-10 | 0.999 |
|  | rs4883723 | 13 | 54084032 | *ZNF646P1, LINC00558* | 1.3e-09 | 2.125E-10 | 6.6165E-09 | 0.000214406 | 3.08521E-05 | 0.999 |
|  | rs12444979 | 16 | 19933600 | *GPRC5B, GPR139* | 4.7e-11 | 8.028E-10 | 1.08412E-09 | 0.000433806 | 2.49799E-06 | 0.999 |
|  | rs1421085 | 16 | 53800954 | *FTO* | 1.3e-102 | 3.201E-19 | 1.4347E-29 | 4.49347E-09 | 3.19286E-21 | 0.999 |
|  | rs6567160 | 18 | 57829135 | *PMAIP1-MC4R* | 5.8e-38 | 4.062E-12 | 8.39552E-26 | 2.62946E-05 | 3.19278E-21 | 0.999 |
| Waist-to-hip ratio | rs2815749 | 1 | 72814783 | *NEGR1* | 7.6e-06 | 1.56E-8 | 7.48039E-05 | 0.002016259 | 0.035698355 | 0.962 |
|  | rs543874 | 1 | 177889480 | *BRINP2, SEC16B* | 5.1e-07 | 2.376E-17 | 2.3403E-10 | 4.41253E-08 | 0.005275775 | 0.995 |
|  | rs6743060 | 2 | 629510 | *FAM150B, TMEM18* | 1.5e-08 | 2.372E-17 | 1.85463E-11 | 4.43012E-08 | 0.000418465 | 0.999 |
|  | rs13130484 | 4 | 45175691 | *PRDX4P1, PRKRIRP9* | 1.5e-08 | 8.936E-11 | 5.67987E-08 | 0.000135674 | 0.000418409 | 0.999 |
|  | rs734597 | 6 | 50836279 | *TFAP2B* | 4.1e-08 | 9.15E-12 | 3.49903E-08 | 4.05196E-05 | 0.000862759 | 0.999 |
|  | rs17309874 | 11 | 27667236 | *BDNF-AS* | 8.1e-08 | 3.217E-8 | 4.31684E-06 | 0.003061711 | 0.001403642 | 0.996 |
|  | rs7138803 | 12 | 50247468 | *NCKAP5L* | 1.2e-05 | 4.978E-18 | 9.49559E-10 | 1.84208E-08 | 0.049021167 | 0.951 |
|  | rs1421085 | 16 | 53800954 | *FTO* | 4.3e-38 | 3.201E-19 | 2.03837E-28 | 4.52217E-09 | 4.5075E-20 | 0.999 |
|  | rs6567160 | 18 | 57829135 | *PMAIP1-MC4R* | 3.1e-10 | 4.062E-12 | 6.75803E-10 | 2.63675E-05 | 2.56288E-05 | 0.999 |

**Additional File 5.** Genes whose expression levels were significantly associated with SNP rs12446632.

| **Gene** | **Tissue** | **P-Value** |
| --- | --- | --- |
| *KNOP1* | Adipose - Subcutaneous | 2.40E-20 |
| *KNOP1* | Adipose - Visceral (Omentum) | 3.70E-24 |
| *KNOP1* | Adrenal Gland | 1.30E-08 |
| *KNOP1* | Artery - Aorta | 3.40E-22 |
| *KNOP1* | Artery - Coronary | 1.20E-10 |
| *KNOP1* | Artery - Tibial | 1.20E-11 |
| *KNOP1* | Brain - Cerebellar Hemisphere | 7.1E-06 |
| *KNOP1* | Brain - Cerebellum | 1.80E-11 |
| *IQCK* | Brain - Cerebellum | 0.000014 |
| *KNOP1* | Brain - Cortex | 4.5E-06 |
| *KNOP1* | Brain - Hypothalamus | 2.60E-09 |
| *KNOP1* | Brain - Nucleus accumbens (basal ganglia) | 4.30E-09 |
| *KNOP1* | Breast - Mammary Tissue | 4.10E-12 |
| *KNOP1* | Cells - EBV-transformed lymphocytes | 1.10E-07 |
| *KNOP1* | Colon - Sigmoid | 3.00E-12 |
| *KNOP1* | Colon - Transverse | 4.20E-10 |
| *KNOP1* | Esophagus - Gastroesophageal Junction | 1.10E-17 |
| *KNOP1* | Esophagus - Mucosa | 1.60E-23 |
| *KNOP1* | Esophagus - Muscularis | 1.60E-28 |
| *KNOP1* | Heart - Atrial Appendage | 1.30E-18 |
| *KNOP1* | Heart - Left Ventricle | 9.50E-14 |
| *KNOP1* | Liver | 7.80E-07 |
| *KNOP1* | Lung | 2.60E-29 |
| *GPRC5B* | Lung | 7.00E-07 |
| *KNOP1* | Muscle - Skeletal | 1.10E-25 |
| *KNOP1* | Nerve - Tibial | 6.10E-24 |
| *KNOP1* | Ovary | 1.60E-10 |
| *KNOP1* | Pancreas | 5.00E-13 |
| *KNOP1* | Pituitary | 1.80E-11 |
| *KNOP1* | Skin - Not Sun Exposed (Suprapubic) | 8.10E-24 |
| *KNOP1* | Skin - Sun Exposed (Lower leg) | 2.60E-29 |
| *KNOP1* | Small Intestine - Terminal Ileum | 1.80E-09 |
| *KNOP1* | Spleen | 1.50E-09 |
| *KNOP1* | Stomach | 4.10E-20 |
| *KNOP1* | Testis | 6.90E-14 |
| *KNOP1* | Thyroid | 6.90E-19 |
| *KNOP1* | Vagina | 1.20E-08 |
| *KNOP1* | Whole Blood | 1.00E-09 |

**Additional File 6.** Canonical pathways significantly enriched in genes associated with childhood BMI-adult cardiometabolic traits.

| **Ingenuity Canonical Pathways** | **Ratio^*^** | **P-value^#^** | **-log(p-value)** | **Molecules** |
| --- | --- | --- | --- | --- |
| Molecular Mechanisms of Cancer | 0.013 | 6.14E-07 | 4.18 | *NFKB1, ADCY3, GNAT2, PRKD1, PMAIP1* |
| Thrombin Signaling | 0.020 | 6.93E-07 | 4.13 | *NFKB1, ADCY3, GNAT2, PRKD1* |
| IL-1 Signaling | 0.033 | 1.47E-06 | 3.83 | *NFKB1, ADCY3, GNAT2* |
| Androgen Signaling | 0.027 | 2.81E-06 | 3.59 | *NFKB1, GNAT2, PRKD1* |
| Corticotropin Releasing Hormone Signaling | 0.027 | 2.97E-06 | 3.57 | *ADCY3, PRKD1, BDNF* |
| Renin-Angiotensin Signaling | 0.025 | 3.95E-06 | 3.47 | *NFKB1, ADCY3, PRKD1* |
| P2Y Purigenic Receptor Signaling Pathway | 0.022 | 5.62E-06 | 3.35 | *NFKB1, ADCY3, PRKD1* |
| GNRH Signaling | 0.021 | 7.38E-06 | 3.26 | *NFKB1, ADCY3, PRKD1* |
| Relaxin Signaling | 0.019 | 1.04E-05 | 3.15 | *NFKB1, ADCY3, GNAT2* |
| Hepatic Cholestasis | 0.019 | 1.11E-05 | 3.13 | *NFKB1, ADCY3, PRKD1* |
| CXCR4 Signaling | 0.018 | 1.26E-05 | 3.09 | *ADCY3, GNAT2, PRKD1* |
| Tec Kinase Signaling | 0.018 | 1.44E-05 | 3.05 | *NFKB1, GNAT2, PRKD1* |
| CREB Signaling in Neurons | 0.016 | 2.07E-05 | 2.94 | *ADCY3, GNAT2, PRKD1* |
| RAR Activation | 0.016 | 2.30E-05 | 2.91 | *NFKB1, ADCY3, PRKD1* |
| Endothelin-1 Signaling | 0.016 | 2.30E-05 | 2.91 | *ADCY3, GNAT2, PRKD1* |
| Phospholipase C Signaling | 0.012 | 6.56E-05 | 2.62 | *NFKB1, ADCY3, PRKD1* |

#overlap ratio represents a ratio of the number of genes from our data set that map to the pathway divide by the total number of molecules that map to the canonical pathway

$ P-values from Fisher’s exact test

**Additional File 7**. Significantly over-represented disease and drug ontologies in the set of genes associated with childhood BMI-adult cardiometabolic traits.

| **ID** | **Name** | **#Genes** | **Genes** | **P-value** | **FDR** |
| --- | --- | --- | --- | --- | --- |
| **Disease ontologies** | | | | | |
| [umls:C0005910](http://www.webgestalt.org/results/Project_wg_result1530917221/Report_wg_result1530917221.html#umls:C0005910) | Body weight | 5 | *MC4R, BDNF, TFAP2B, FTO, SEC16B* | 1x10^-11^ | 1.5 x10^-8^ |
| [umls:C0028754](http://www.webgestalt.org/results/Project_wg_result1530917221/Report_wg_result1530917221.html#umls:C0028754) | Obesity | 7 | *TMEM18, NEGR1, GNAT2, MC4R, BDNF, TFAP2B, FTO* | 1.61x10^-7^ | 1.21 x10^-4^ |
| [umls:C0036341](http://www.webgestalt.org/results/Project_wg_result1530917221/Report_wg_result1530917221.html#umls:C0036341) | Schizophrenia | 5 | *MC4R, NFKB1, GALNT10, PRKD1, BDNF* | 5.45x10^-5^ | 2.73 x10^-2^ |
| **Drug ontologies** | | | | | |
| [PA164712878](http://www.webgestalt.org/results/Project_wg_result1530916563/Report_wg_result1530916563.html#PA164712878) | Low-energy diets | 3 | *TFAP2B, FTO, MC4R* | 2.26x10^-5^ | 2.93x10^-2^ |

**References**

1. Felix JF, Bradfield JP, Monnereau C, van der Valk RJ, Stergiakouli E, Chesi A, et al. Genome-wide association analysis identifies three new susceptibility loci for childhood body mass index. Hum Mol Genet. 2016;25(2):389-403.

2. Locke AE, Kahali B, Berndt SI, Justice AE, Pers TH, Day FR, et al. Genetic studies of body mass index yield new insights for obesity biology. Nature. 2015;518(7538):197-206.

3. Shungin D, Winkler TW, Croteau-Chonka DC, Ferreira T, Locke AE, Magi R, et al. New genetic loci link adipose and insulin biology to body fat distribution. Nature. 2015;518(7538):187-96.

4. Replication DIG, Meta-analysis C, Asian Genetic Epidemiology Network Type 2 Diabetes C, South Asian Type 2 Diabetes C, Mexican American Type 2 Diabetes C, Type 2 Diabetes Genetic Exploration by Nex-generation sequencing in muylti-Ethnic Samples C, et al. Genome-wide trans-ancestry meta-analysis provides insight into the genetic architecture of type 2 diabetes susceptibility. Nat Genet. 2014;46(3):234-44.

5. Dupuis J, Langenberg C, Prokopenko I, Saxena R, Soranzo N, Jackson AU, et al. New genetic loci implicated in fasting glucose homeostasis and their impact on type 2 diabetes risk. Nat Genet. 2010;42(2):105-16.

6. Soranzo N, Sanna S, Wheeler E, Gieger C, Radke D, Dupuis J, et al. Common variants at 10 genomic loci influence hemoglobin A(1)(C) levels via glycemic and nonglycemic pathways. Diabetes. 2010;59(12):3229-39.

7. Prokopenko I, Poon W, Magi R, Prasad BR, Salehi SA, Almgren P, et al. A central role for GRB10 in regulation of islet function in man. PLoS Genet. 2014;10(4):e1004235.

8. Walford GA, Gustafsson S, Rybin D, Stancakova A, Chen H, Liu CT, et al. Genome-Wide Association Study of the Modified Stumvoll Insulin Sensitivity Index Identifies BCL2 and FAM19A2 as Novel Insulin Sensitivity Loci. Diabetes. 2016;65(10):3200-11.

9. Nikpay M, Goel A, Won HH, Hall LM, Willenborg C, Kanoni S, et al. A comprehensive 1,000 Genomes-based genome-wide association meta-analysis of coronary artery disease. Nat Genet. 2015;47(10):1121-30.

10. Willer CJ, Schmidt EM, Sengupta S, Peloso GM, Gustafsson S, Kanoni S, et al. Discovery and refinement of loci associated with lipid levels. Nat Genet. 2013;45(11):1274-83.
